# A New Weak Chelator in Endodontics: Effects of Different Irrigation Regimens with Etidronate on Root Dentin Microhardness

**DOI:** 10.1155/2013/743018

**Published:** 2013-07-25

**Authors:** Talita Tartari, Patrícia de Almeida Rodrigues Silva e Souza, Bruno Vila Nova de Almeida, José Otávio Carrera Silva Júnior, Oscar Facíola Pessoa, Mario Honorato Silva e Souza Junior

**Affiliations:** ^1^Dental School, Federal University of Pará, 66075-900 Belém, PA, Brazil; ^2^Department of Endodontics, Dental School, Federal University of Pará, 66075-900 Belém, PA, Brazil; ^3^Pharmacy School, Federal University of Pará, 66075-900 Belém, PA, Brazil; ^4^Department of Restorative Dentistry, Dental School, Federal University of Pará, 66075-900 Belém, PA, Brazil

## Abstract

This study investigated the effect of sodium hypochlorite (NaOCl), ethylenediaminetetraacetic (EDTA), etidronic (HEBP), and citric acid (CA) associated in different irrigation regimens on root dentin microhardness. Forty-five root halves of single-rooted teeth were sectioned into thirds that were embedded in acrylic resin, polished, randomly assigned into 3 groups, and treated as follows: G1: saline solution; G2: 5% NaOCl + 18% HEBP, mixed in equal parts; and G3: 2.5% NaOCl. After measurements, the G3 samples were distributed into subgroups G4, G5, and G6, which were submitted to 17% EDTA, 10% CA and 9% HEBP, respectively. Following the new measurements, these groups received a final flush with 2.5% NaOCl, producing G7, G8, and G9. Microhardness was measured with Knoop indenter under a 25 g load for 15 seconds, before and after treatments. The data were statistically analyzed using paired Student's *t*-test (α<0.05) to compare values before and after treatments and analysis of variance (ANOVA) (α<0.05) to detect any differences among thirds. Except G1, all tested irrigation regimens significantly decreased the microhardness. There were no differences between root thirds before treatments, and all root thirds exhibited equal responses to same treatment. Except saline, all tested irrigation regimens reduced the root dentin microhardness.

## 1. Introduction

During endodontic therapy, chemical solutions are used to assist the action of endodontic instruments in the process of cleaning and shaping the root canal system. The lubrication of dentinal walls by these solutions would lower mechanical stress on rotary root canal instruments preventing instrument separation [1]. 

Despite the fact that these solutions facilitate the root canal instrumentation, they can also increase the possibility of occurrence of root canal deviation during biomechanical preparation [2], because they interfere in chemical structure of dentin, modifying the calcium/phosphorus (Ca/P) ratio of the surface [3, 4], which may decrease microhardness [5] facilitating the dentin cutting. These changes can also affect the sealing ability and adhesion of root canal sealers [6, 7]. Studies indicated that sodium hypochlorite (NaOCl), the most commonly used irrigating agent, decreases the microhardness of dentin at all concentrations [8–10]. With regard to chelating agents, the decalcification effects depend heavily on the irrigant used, the application time, and the solution pH and concentration [11, 12].

The association of different solutions with capability to dissolve organic and inorganic components is necessary for the complete removal of the smear layer and disruption of the bacterial biofilm [5, 13]. Currently, some studies showed that when a chelator, such as ethylenediaminetetraacetic acid (EDTA), citric acid (CA), and MTAD (mixture of doxycycline, citric acid, and Tween 80), is used as a final irrigant, the collagen matrix remains on the surface of the root canal [14, 15], which may contribute to bacterial adherence in recontaminations [16, 17]. Therefore, to minimize this effect and restore the surface characteristics of untreated dentin, the use of NaOCl solutions has been suggested after the use of chelating agents [17] to remove this exposed collagen matrix in a process called deproteination [3].

Recently, the hydroxyethylidene bisphosphonate (HEBP), also known as etidronate, a substance that prevents bone resorption and is used systemically in patients suffering from osteoporosis or Paget's disease [18, 19] has been suggested as a substitute for other chelators because it has fewer effects on the dentin structure [20] and can even be mixed with NaOCl solution without interfering with the antimicrobial property of this substance [21]. However, these solutions need 300 seconds to completely remove the *smear layer* [20]. 

Although several studies have evaluated the effect of irrigating solutions on root dentin microhardness [5, 8, 12, 22], additional studies are necessary to analyze the effect of different irrigation regimens employing NaOCl isolated and associated with different chelating agents followed or not by a final flush with NaOCl, because the reduction in microhardness could be potentiated by combination of these solutions. Consequently, the aim of this study was to evaluate the effect of the NaOCl, EDTA, HEBP, and CA associated in different irrigation regimens on root dentin microhardness.

## 2. Methods

This study was reviewed and approved by the Ethics Committee on Human Research of the Health Sciences Institute of Federal University of Pará, Brazil (CEP-ICS/UFPA; Protocol no. 134/11).

### 2.1. Solutions

The following solutions were used in the irrigation regimens: 2.5% (wt/vol) and 5% NaOCl, 17% EDTA, 10% CA, and 9 and 18% HEBP. The 17% EDTA solution was prepared by dissolving disodium EDTA (Sigma Aldrich, St. Louis, MO, USA) in distilled water with the aid of sodium hydroxide (Sigma Aldrich) to favor dissolution; the pH was adjusted to 7 by adding hydrochloric acid (HCl; Sigma Aldrich). The 2.5 and 5% NaOCl, 10% CA (Sigma Aldrich), and 9 and 18% HEBP (Zschimmer & Schwarz Mohsdorf GmbH & Co KG, Burgstädt, Germany) solutions were prepared by mixing pure chemicals with distilled water.

All solutions were stored in dark containers at 5°C between experiments; prior to being used, they were removed from the refrigerator and stored for 60 min at room temperature. To obtain the solution with 2.5% NaOCl and 9% HEBP, a fresh 1 : 1 mixture of 5% NaOCl and 18% HEBP was prepared immediately before the experiments [21].

### 2.2. Tooth Selection and Specimen Preparation

Single-rooted human teeth were selected based on their relative dimensions, similarity in morphology and the absence of any cracks or caries defects within root portions. Tissue and debris remnants on the root surfaces were removed, and all teeth were stored in 0.1% thymol at 9°C until use.

The teeth were decoronated at the cementum-enamel junction using a low-speed diamond disk (KG Sorensen Ind. e Com., São Paulo, SP, Brazil) under coolant water. Sectioning was performed again at the level of apical foramen to ensure that only the canal dentin was analyzed. Each root was then bisected longitudinally to obtain the 45 root halves needed for the study. The sample size was determined after a pilot study. Then, the halves were horizontally sectioned into apical, middle, and cervical thirds, being the thirds previously marked with the aid of a digital caliper PD-150 (Vonder, Curitiba, PR, Brazil). The specimen segments were identified and embedded in autopolymerizing acrylic resin (Dent Bras, Pirassununga, SP, Brazil), leaving the root canal dentin exposed. 

The dentin was prepared for microhardness tests by polishing the surface on a circular grinding machine with a series of ascending grades (400, 600, 1200, and 2000) of silicon carbide abrasive papers (3M, Sumaré, SP, Brazil) under constant water irrigation in a polishing machine (Arotec, São Paulo, SP, Brazil). Then, the specimens were polished with a felt disc and extra-fine-grained diamond paste (Diamond Excel, FGM Dental Products, Joinville, SC, Brazil). Afterward, the specimens were rinsed and ultrasonicated in distilled water for 5 min to remove any residue.

The reference microhardness values of untreated specimens were recorded using a Knoop indenter of the Microhardness Tester FM-700 (Future-Tech, Kawasaki, Japan), employing a 25 g load and a 15-second dwell time. In each sample, three indentations were made along the root canal lumen following a straight line toward one adjacent to each other. The mean of these values was used to compare alterations in microhardness. 

The samples were then randomly distributed into groups as follows: G1 (*n* = 9): saline solution (control) for 30 min; G2 (*n* = 9): 5% NaOCl + 18% HEBP, mixed in equal parts for 30 min; and G3 (*n* = 27): 2.5% NaOCl for 30 min. After the microhardness measurements, the G3 samples were divided to form G4, G5, and G6 (*n* = 9), which received the following chelating agents to remove the smear layer: 17% EDTA for 3 min, 10% CA for 3 min, and 9% HEBP for 5 min, respectively. Following the new microhardness measurements, the samples in Groups G4, G5, and G6 received a final flush with 2.5% NaOCl for 3 min to remove the exposed collagen matrix by chelation, resulting in Groups G7, G8, and G9.

In each step, the specimens were immersed in 40 mL of the test solutions and ultrasonicated. The irrigation solutions were changed every 5 min to ensure their chemical effectiveness. To avoid the prolonged effect of solutions, the specimens received a final flush for 1 min with 40 mL of distilled water in an ultrasonic tub.

The posttreatment indentations were made on each specimen, adjacent to the initial indentations and made in the same manner; the microhardness values were then recorded.

### 2.3. Statistical Analysis

The sample size was determined after a pilot study. For this calculation, it was taken into account that after the first treatment the mean microhardness was 37.9 and the standard deviation of differences was 3.52. To detect a difference of 10%, using a paired *t*-test power of 80% and a bilateral *α* of 5%, the required sample size was 9 specimens per group. 

Twenty-seven specimens of each root third were subjected to NaOCl for 30 min, and they were later distributed according to the irrigation regimens. To avoid false-positive inflation, the microhardness values of only 9 specimens were randomly selected for statistical analysis of the G3. 

The sample showed normal distribution. The Student's *t*-test was used to compare the dentin surface microhardness before and after treatments, and analysis of variance (ANOVA) and Tukey's multiple-comparison was used to detect any differences among the root thirds. All hypothesis testing was performed at a 95% confidence level.

## 3. Results

The mean and standard deviation in microhardness of the cervical, middle, and apical thirds of the root canal lumen dentin in the different experimental groups, both before and after treatments, are shown in Tables 1, 2, and 3. It was observed that, except control group, all tested irrigation regimens significantly reduced the microhardness (*P* < 0.05). 

A comparison of the different thirds revealed no statistically significant differences in their initial microhardness values. Analysing the behavior of root thirds is possible to affirm that the different thirds behave in the same way when submitted to the same treatment (Table 4).

## 4. Discussion

A reduction in dentin microhardness caused by irrigating solutions used in endodontic treatments not only can facilitate the preparation of narrow or calcified canals, but also it may increase the chances of canal deviation to occur [2, 23].

The results show that, except in G1 (control), all tested irrigation regimens significantly decreased the dentin microhardness, confirming the results of studies that indicate that these irrigating solutions interfere with the chemical composition of the dentin surface [3, 4].

For a given load, the Vickers indenter penetrates approximately twice as far into the specimen as the more shallow Knoop indenter [24]. Furthermore, the hardness measurements obtained by the Knoop method are practically insensitive to the elastic recovery of the material, which made this test much more appropriate for the analysis of surface microhardness [25]. These characteristics suggest that superficial dentin, closer to the pulp, should be analyzed with this method; for these reasons, the Knoop test was chosen for this study.

Sodium hypochlorite can dissolve proteins, and if used after the application of a chelating solution, it can optimize surfaces for the adhesion of materials based on its reaction with the mineral phase of dentin [4]; it also reduces the adhesion of bacteria to exposed collagen on the surface by calcium-complexing agents [16, 17]. The results showed that NaOCl is able to significantly decrease the dentin microhardness, even when used alone (G3), which agrees with previous research [8–10]. When used in the final rinsing for only 3 minutes, this solution does not significantly change the microhardness compared with the values in the groups that did not have a final flush with NaOCl, confirming that its effect is time dependent [8].

In the experiment, after the use of chelators, the smear layer and plugs were removed, and dentinal tubule orifices could be seen in the specimens surface. The irrigation regimes that employed HEBP as a chelating agent resulted in significant decreases in hardness, but these values were lower than those resulting from protocols that employed CA or EDTA (*P* < 0.001) (Tables 1, 2, and 3). It shows that the decrease in hardness is directly proportional to the chelating power of the substance. These findings confirm that HEBP is a weak calcium-complexing agent that causes less change in dentin than other chelating agents [6, 12, 20]. 

Despite that the root thirds are structurally different [26–28], a comparison of initial values showed that the surface microhardness is similar between them. The same result was observed after the use of irrigating solutions. When subjected to the same irrigation regimen, the thirds behaved similarly, proving that when the irrigating solution comes into direct contact with the dentin surface, although the structure is different in each region, the resulting alterations are similar.

It is possible to affirm that except saline, all tested irrigation regimens reduced the microhardness of the root dentin lumen, but these results should not be extrapolated to clinical practice, because during an endodontic therapy, instruments are also used, and it is difficult to bring irrigation solutions close to the apex region, which could influence the recorded values. The root thirds are structurally different, so the analysis of the behavior of these thirds into direct contact with the irrigating solutions is necessary to know if besides the difficulty of irrigators to reach the apex, the dentin composition also influences the decrease of microhardness. More studies are needed to evaluate not only the effect of different irrigation regimes on the dentin structure, but also the effect of these protocols on the adhesion of bacteria and root canal fillings.

## 5. Conclusions

Based on the results of this study, the following can be concluded.

1. Except saline, all tested irrigation regimens reduced the microhardness of human root lumen canal dentin.
2. Despite being structurally different, when subjected to the same irrigation regimen, the root thirds behaved similarly.

## Figures and Tables

**Table 1:** Mean and standard deviation and *P* values (Student's *t-*test) for microhardness analysis in the cervical third before (T0) and after (T1) the application of the irrigation regimens.

Groups	Cervical third (CT)		
	T0 X ± SD	T1 X ± SD	*P* value (Student's *t-*test)
G1: saline	46.6 ± 6.3	46.0 ± 5.2	0.69
G2: mixture 5% NaOCl and 18% HEBP (30 min)	43.7 ± 5.0	36.2 ± 5.4	0.02
G3: 2.5% NaOCl (30 min)	44.7 ± 3.5	38.7 ± 3.8	0.02
G4: 2.5% NaOCl (30 min) + 17% EDTA (3 min)	47.5 ± 6.4	30.7 ± 3.5	<0.0001
G5: 2.5% NaOCl (30 min) + 10% CA (3 min)	43.7 ± 3.4	31.5 ± 4.9	<0.0001
G6: 2.5% NaOCl (30 min) + 9% HEBP (5 min)	45.9 ± 4.8	41.4 ± 4.9	0.04
G7: 2.5% NaOCl (30 min) + 17% EDTA (3 min) + 2.5% NaOCl (3 min)	47.5 ± 6.4	30.2 ± 3.91	<0.0001
G8: 2.5% NaOCl (30 min) + 10% CA (3 min) + 2.5% NaOCl (3 min)	43.7 ± 1.8	31.9 ± 6.8	<0.0001
G9: 2.5% NaOCl (30 min) + 9% HEBP (5 min) + 2.5% NaOCl (3 min)	45.9 ± 4.8	39.1 ± 4.76	0.02

X: mean; SD: standard deviation.

**Table 2:** Mean and standard deviation and *P* values (Student's *t-*test) for microhardness analysis in the middle third before (T0) and after (T1) the application of the irrigation regimens.

Groups	Middle third (MT)		
	T0 X ± SD	T1 X ± SD	*P* value (Student's *t-*test)
G1: saline	46.9 ± 5.1	45.1 ± 3.7	0.43
G2: mixture 5% NaOCl and 18% HEBP (30 min)	45.5 ± 5.5	35.7 ± 4.1	0.006
G3: 2.5% NaOCl (30 min)	44.9 ± 5.0	39.8 ± 2.9	0.01
G4: 2.5% NaOCl (30 min) + 17% EDTA (3 min)	47.3 ± 3.7	34.5 ± 5.4	<0.001
G5: 2.5% NaOCl (30 min) + 10% CA (3 min)	45.2 ± 3.5	31.4 ± 7.4	<0.0001
G6: 2.5% NaOCl (30 min) + 9% HEBP (5 min)	47.7 ± 4.6	42.6 ± 3.0	0.02
G7: 2.5% NaOCl (30 min) + 17% EDTA (3 min) + 2.5% NaOCl (3 min)	47.3 ± 3.7	34.4 ± 5.4	<0.0001
G8: 2.5% NaOCl (30 min) + 10% CA (3 min) + 2.5% NaOCl (3 min)	45.6 ± 2.9	29.8 ± 6.4	<0.0001
G9: 2.5% NaOCl (30 min) + 9% HEBP (5 min) + 2.5% NaOCl (3 min)	47.7 ± 4.6	41.8 ± 4.2	0.004

X: mean; SD: standard deviation.

**Table 3:** Mean and standard deviation and *P* values (Student's *t-*test) for microhardness analysis in the apical third before (T0) and after (T1) the application of the irrigation regimens.

Groups	Apical third (AT)		
	T0 X ± SD	T1 X ± SD	*P* value (Student's *t-*test)
G1: saline	47.9 ± 6.8	43.7 ± 7.3	0.21
G2: mixture 5% NaOCl and 18% HEBP (30 min)	46.1 ± 3.7	40.0 ± 5.7	0.02
G3: 2.5% NaOCl (30 min)	45.2 ± 2.8	40.7 ± 5.0	0.02
G4: 2.5% NaOCl (30 min) + 17% EDTA (3 min)	47.2 ± 3.6	35.3 ± 4.0	<0.0001
G5: 2.5% NaOCl (30 min) + 10% CA (3 min)	45.4 ± 7.0	30.2 ± 5.4	<0.0032
G6: 2.5% NaOCl (30 min) + 9% HEBP (5 min)	46.4 ± 6.1	39.6 ± 5.8	0.01
G7: 2.5% NaOCl (30 min) + 17% EDTA (3 min) + 2.5% NaOCl (3 min)	47.2 ± 3.6	35.7 ± 5.2	0.0006
G8: 2.5% NaOCl (30 min) + 10% CA (3 min) + 2.5% NaOCl (3 min)	45.1 ± 7.5	28.0 ± 3.6	<0.0005
G9: 2.5% NaOCl (30 min) + 9% HEBP (5 min) + 2.5% NaOCl (3 min)	46.4 ± 6.1	39.4 ± 4.9	0.04

X: mean; SD: standard deviation.

**Table 4:** The *P* values (ANOVA) of the microhardness before (T0) and after (T1) the administration of the same treatment on different thirds.

Groups	CT × MT CT × AT MT × AT *P* value in T0 (ANOVA)	CT × MT CT × AT MT × AT *P* value in T1 (ANOVA)
G1: saline	0.89	0.68
G2: mixture 5% NaOCl and 18% HEBP (30 min)	0.57	0.16
G3: 2.5% NaOCl (30 min)	0.95	0.58
G4: 2.5% NaOCl (30 min) + 17% EDTA (3 min)	0.99	0.08
G5: 2.5% NaOCl (30 min) + 10% CA (3 min)	0.74	0.86
G6: 2.5% NaOCl (30 min) + 9% HEBP (5 min)	0.76	0.58
G7: 2.5% NaOCl (30 min) + 17% EDTA (3 min) + 2.5% NaOCl (3 min)	0.99	0.06
G8: 2.5% NaOCl (30 min) + 10% CA (3 min) + 2.5% NaOCl (3 min)	0.74	0.61
G9: 2.5% NaOCl (30 min) + 9% HEBP (5 min) + 2.5% NaOCl (3 min)	0.76	0.59

CT: cervical third; MT: middle third; AT: apical third.
